# Investigation of Spatial Distribution of Radiocesium in a Paddy Field as a Potential Sink

**DOI:** 10.1371/journal.pone.0080794

**Published:** 2013-11-15

**Authors:** Kazuya Tanaka, Hokuto Iwatani, Yoshio Takahashi, Aya Sakaguchi, Kazuya Yoshimura, Yuichi Onda

**Affiliations:** 1 Institute for Sustainable Sciences and Development, Hiroshima University, Kagamiyama, Higashi-Hiroshima, Hiroshima, Japan; 2 Department of Earth and Planetary Systems Science, Graduate School of Science, Hiroshima University, Kagamiyama, Higashi-Hiroshima, Hiroshima, Japan; 3 Center for Research in Isotopes and Environmental Dynamics, University of Tsukuba, Tennodai, Tsukuba, Ibaraki, Japan; Dowling College, United States of America

## Abstract

Surface soils, under various land uses, were contaminated by radionuclides that were released by the Fukushima Daiichi Nuclear Power Plant accident. Because paddy fields are one of the main land uses in Japan, we investigated the spatial distribution of radiocesium and the influence of irrigation water in a paddy field during cultivation. Soil core samples collected at a paddy field in Fukushima showed that plowing had disturbed the original depth distribution of radiocesium. The horizontal distribution of radiocesium did not show any evidence for significant influence of radiocesium from irrigation water, and its accumulation within the paddy field, since the original amount of radiocesium was much larger than was added into the paddy field by irrigation water. However, it is possible that rainfall significantly increases the loading of radiocesium.

## Introduction

 Huge amounts of radionuclides, especially ^134^Cs, ^137^Cs and ^131^I, were released into the environment by the Fukushima Daiichi Nuclear Power Plant (FDNPP) accident. Chino et al. [1] estimated that the total amounts of ^131^I and ^137^Cs discharged into the atmosphere between 10:00 JST on March 12 and 00:00 JST on April 6 were approximately 1.5 × 10^17^ and 1.3 × 10^16^ Bq, respectively. The migration of radiocesium from land area to ocean through river systems was previously investigated in Fukushima [2]. In the migration process, paddy fields can be one of the main sinks or sources of radiocesium, since they are one of the main land uses in the Fukushima area. Thus, the budget of radiocesium during the cultivation of rice in paddy fields is of great interest, especially because cultivation of rice draws a large amount of pond or riverine water and also discharges a large amount of irrigation water from the paddy fields. As rice is the staple food of Japanese people, it is therefore important to evaluate the possible migration of radiocesium from contaminated soils and water into rice. 

**Figure 1:**
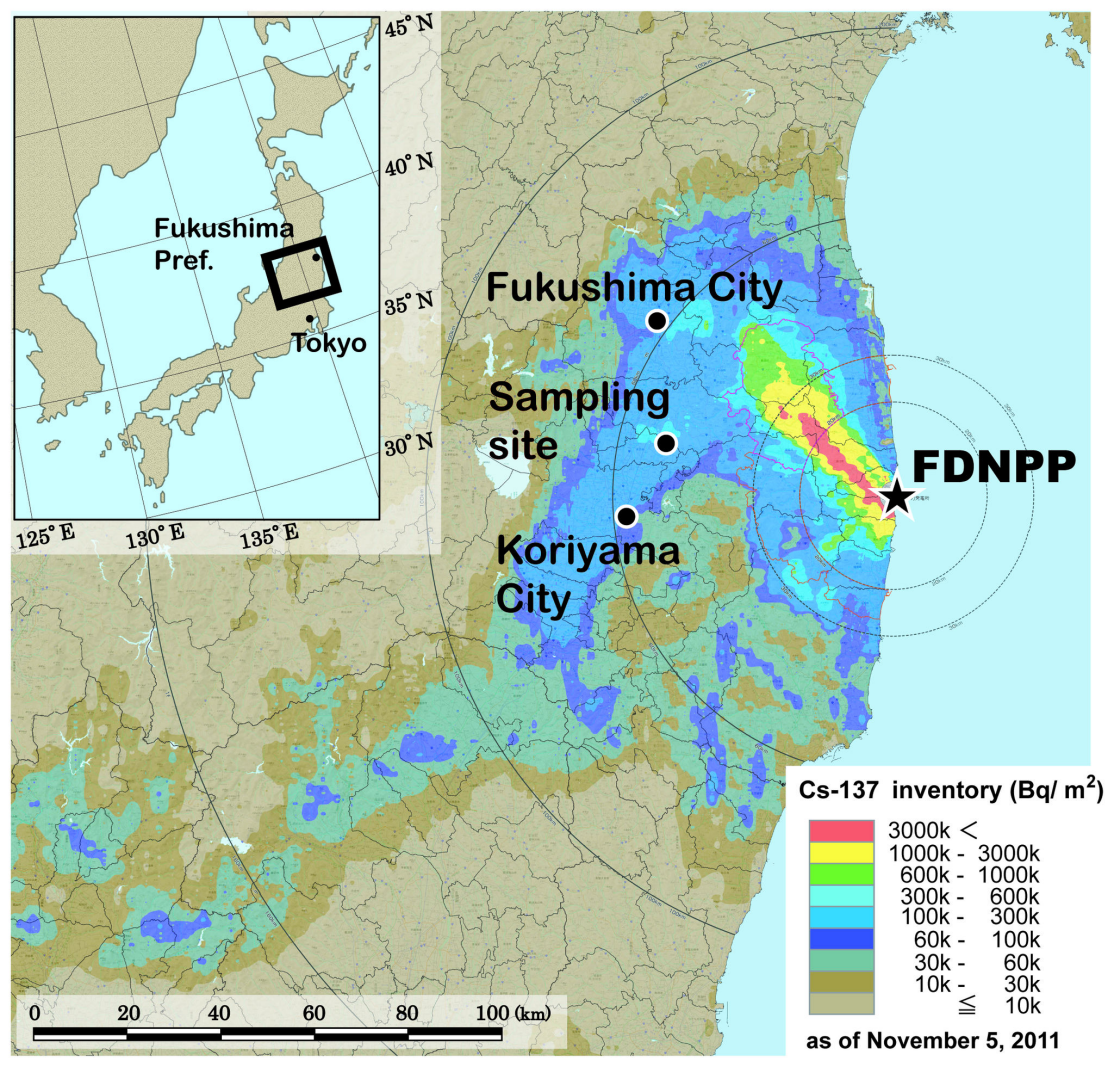
Sampling site of paddy soils plotted on the map of ^137^Cs inventory around the FDNPP estimated by aircraft monitoring as of November 5, 2011[6].

 The ^137^Cs inventories of global fallout, originating from atmospheric nuclear tests in the 1960s, have been estimated at ca. 2 - 5 kBq/m^2^ in Japan [3-5]. The current inventories of ^137^Cs emitted by the FDNPP accident in Fukushima Prefecture are much higher than those originating from nuclear tests (Figure 1). Consequently, primary contamination of paddy fields in Fukushima can be attributed to fallout of radiocesium by the FDNPP accident. The spatial distribution of radiocesium can be drastically changed during cultivation. In addition, it is possible that input of contaminated soil particles and water into paddy fields from surrounding environments causes further contamination, leading to the accumulation of radiocesium and heavier contamination with time. Irrigation water can be a main carrier of such secondary input of radiocesium. In this study, therefore, we investigated the load of radiocesium input, relative to the initial fallout from the FDNPP accident, at a paddy field in Fukushima.

## Samples and Methods

### Rice cultivation

 In central Japan, the rice cultivation season is usually from around May to September. In the process of rice cultivation, the discharges of water from paddy fields are only two times. The first time is during the plowing event when irrigation is conducted while planting rice seedlings around May. The second time is about two weeks before the harvest at the end of September. Through the cultivation period, a paddy field is water laden with a depth of 3 to 5 cm. Normally, both the inlet and outlet of the paddy field are closed, and water does not pass through the paddy. Thus, all the suspended solids, if irrigation water is further drawn from the inlet, can be deposited in the paddy field.

### Paddy soils and irrigation water

 Soil and irrigation water samples were collected at a paddy field (area 740 m^2^) with permission of the owner, on August 31 or September 16, 2012 at Motomiya City in Fukushima Prefecture. The sampling dates correspond to the late stage of cultivation. The sampling site is located 50 km west-northwest of the FDNPP (Figure 1). The inventory around the sampling site of ^137^Cs originating from the FDNPP accident was roughly estimated at 300 - 600 kBq/m^2^ based on aircraft monitoring as of November 5, 2011 (Figure 1) [6]. After the FDNPP accident, rice was cropped, accompanied with plowing, in the paddy field in both 2011 and 2012. Therefore, soil layers were mixed, to some extent, both vertically and horizontally. The irrigation water was drawn from an irrigation pond about 50 m away from the paddy field.

**Figure 2:**
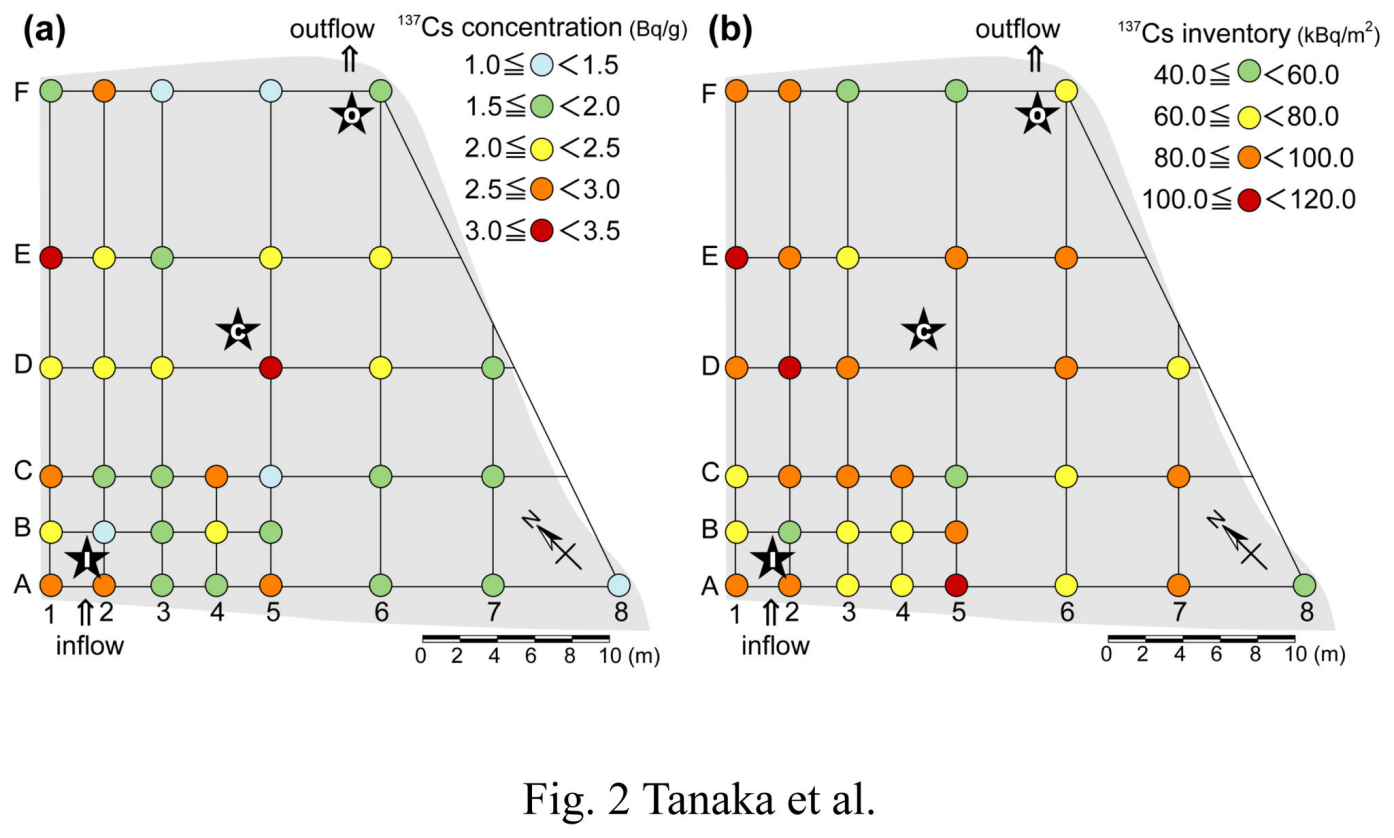
Sampling points of 5 cm and 30 cm soil core samples in the paddy field. The stars indicate the sampling points of the 30 cm soil cores. Horizontal distribution of (a) ^137^Cs concentrations and (b) inventories in the uppermost 5 cm soil layers are plotted in a color scale.

 At the time of the sampling, the soil was watery and muddy although the surface of the soil was not covered with water. Through an interview with the owner, it was confirmed that water was not discharged from the paddy field after plowing as described above. Possibly, the water level in the paddy field was reduced by evaporation, penetration into soil layers and uptake by the ears of rice. Surface soil up to a depth of 5 cm from the surface was collected at 36 grid locations (Figure 2), using a plastic corer with an inner diameter of 5.0 cm. Soil core samples with a length of 30 cm were also taken at three points: points I and O (labeled MTMC-I and -O) were near the water inlet and outlet, respectively, while point C (labeled MTMC-C) was in the middle of the paddy field (Figure 2). A stainless steel pipe equipped with an inner plastic corer (diameter 5 cm) was inserted into the soil layer at each point. The collected core samples were subsampled at intervals of 2 cm from the surface. Core descriptions, corresponding to each soil layer, are summarized in Table S1. Overall, the paddy soils were characterized by dark gray mud containing humic substances, mica and granules. The surface and subsampled core soils were air-dried, and then homogenized for measurement of radiocesium as described below.

 Irrigation water was collected near the water inlet outside of the paddy field. The irrigation water sample was filtered on site to separate suspended particles. Suspended particles were divided into three fractions of particle size > 63 μm, 3 - 63 μm and 0.45 - 3 μm particles by sieving and filtration. Here, we regarded a fraction passing through a filter of pore size 0.45 μm as a soluble fraction of radiocesium. 

### Monitoring of rainfall, water flow and load of suspended sediments into paddy field

 The study monitored rainfall, water flow rate and concentrations of suspended particles and particulate radiocesium at the inlet head of the paddy field to evaluate the load of suspended particles and radiocesium into the paddy field. Monitoring was carried out from August 23 to September 19, 2012. The rainfall was monitored using a rain gauge (Rain Collector II; Davis Instruments, CA, USA). The flow rate was monitored with a V-notch weir combined with a water level gauge (WT-HR; TruTrack, Christchurch, New Zealand). The concentration of suspended particles in the inflow water was measured with a turbidity probe (ANALITE NEP180/30G, McVan Instruments, Victoria, Australia). Then, sediment load was calculated from the discharge and the concentration of suspended particles. In addition to this monitoring, suspended particles were collected by a time-integrated suspended particle sampler during the monitoring period [7]. The suspended particle sample was dried and disaggregated, followed by the measurement of radiocesium.

### Sample preparation and measurement of ^137^Cs

 Homogenized soil samples were loaded into cylindrical polystyrene containers with an inner diameter of 5.0 cm and height of 6.8 cm. The soil sample, in the container, was placed on a planar-type Ge semiconductor detector (CANBERRA, GC4018/7915-30/ULB-GC, relative efficiency: 45%) to determine the count rate of γ-rays emitted from ^137^Cs (662 keV). Actually, this detector is low-back ground specification, and the integrated count rate on the ROI for ^137^Cs was 1.9 x 10^-4^ cps as detector background. There is no ^137^Cs background on this detector. The dependence of the detection efficiency for 662 keV on the height of the sample in the container was determined using the IAEA reference material of IAEA-444 for conversion of count rates to radioactivity. We set the height of all the samples at 7 mm, for which the detection efficiency was 0.042 cps/Bq. All the samples were measured within l0% error (1 sigma standard deviation from counting statistic) to discuss the ^137^Cs distribution pattern accurately. Counting time varied between 440 and 2,200 s, depending on the radiocesium concentration in the soil samples. The calculated activities were corrected to the sampling dates of August 31 or September 16. 

 Filtered irrigation water sample was measured after preconcentration treatment using ammonium molybdophosphate (AMP) [8]. The stable isotope of ^133^Cs was added to 20 L of the filtered water as a carrier to estimate the recovery rate of the preconcentration process. After acidifying the water sample to pH 1 with HNO_3_, 4 grams of AMP powder were added as an adsorbent for Cs. The Cs-adsorbed AMP powder was dried at 105°C, and then packed into a plastic bag (4.5 cm × 4.5 cm) for measurement of ^137^Cs (662 keV) by γ-ray spectrometry using a planar-type Ge detector (CANBERRA, GC4018/7915-30/ULB-GC). The recovery rate of Cs was 92%, indicating successful treatment of the water sample. The procedure is described in detail in Sakaguchi et al. [8]. Suspended particles collected on filters were packed into a plastic bag, and measured as for the Cs-adsorbed AMP sample. Counting time varied between 260 and 86,000 s for the concentrated water and suspended particle samples.

## Results and Discussion

### Depth distribution of ^137^Cs in soil layers

**Figure 3:**
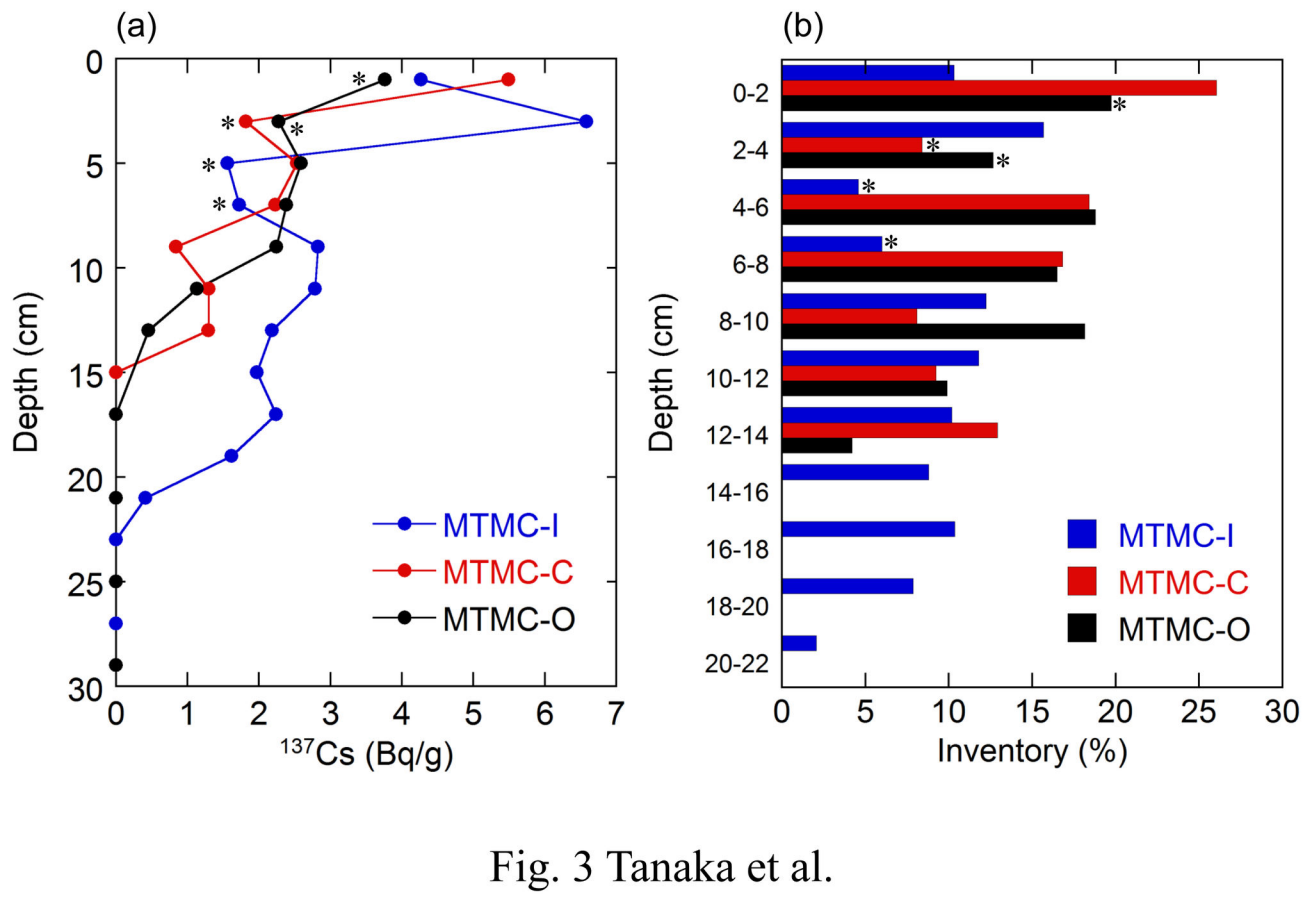
Depth distribution of (a) ^137^Cs concentrations and (b) inventories in soil core profiles. The ^137^Cs concentrations are plotted against the median at each depth interval. The asterisks indicate sand layer.

 The analytical results of ^137^Cs concentrations in surface soil core samples (about 30 cm) are listed in Table S1. Depth distributions of ^137^Cs concentrations and inventories are shown in Figure 3. Overall, three sampling points in the paddy field indicate that ^137^Cs concentrations as well as inventories in the soil layers were the highest at or near the surface, and decreased with depth. The depth distribution of ^137^Cs in the paddy soils is quite different from that observed in undisturbed Fukushima soils. Cesium-137 concentrations and inventories in undisturbed Fukushima soils showed an exponential decrease with depth [9-11]. However, the depth distribution of ^137^Cs concentrations and inventories shows both increases and decreases in the paddy soil layers rather than an exponential decrease (Figure 3). It has been reported that more than 95% of the radiocesium deposited on the ground stayed above 6 cm in soil layers in Fukushima [9-11]. By contrast, 50 - 70% of the ^137^Cs inventories in our 30 cm core samples are observed below 6 cm in depth (Table S1 and Figure 3b). Once radiocesium is adsorbed on soil particles, it is not easily released into water [2,9,12]. In fact, Matsunaga et al. [13] recently reported that the depth profile of ^137^Cs was almost unchanged before and after the rainy season in 2011. The observed characteristics of ^137^Cs distribution clearly indicate mixing of upper and lower soil layers in the paddy field, which is consistent with the fact that plowing of the field was carried out with irrigation for planting rice seedlings. The plowing process possibly sorted soil particles with different sizes, resulting in the accumulation of larger particles and the formation of sand layers at the depth intervals of 4 - 8 cm, 2 - 4 cm and 0 - 4 cm at points I, C and O, respectively (Table S1). Since larger particles have lower ^137^Cs concentration [14], it is reasonable that these sand layers correspond to lower ^137^Cs concentration and inventory than the layers above and below (Figure 3).

 The total inventories of ^137^Cs in the soil layers were calculated at 503, 248 and 293 kBq/m^2^ for points I, C and O, respectively (Table S1). The average of the inventories is 348 kBq/m^2^, which is within the range estimated from the radiocesium map by MEXT [6] (Figure 1). The variation of the ^137^Cs inventories observed in the same paddy field indicates the heterogeneous distribution of fallout radiocesium from the FDNPP accident. Ohno et al. [11] also reported similar heterogeneity of inventories for radiocesium and radioiodine in different agricultural fields. Such field-scale variation of inventories was attributed to the heterogeneity of radiocesium at a microscale [2]. 

### Horizontal distribution of ^137^Cs in a paddy field

 The analytical results of ^137^Cs concentrations in surface soil samples within 5 cm of the surface are listed in Table S2. Cesium-137 concentration in the surface soils varied between 1.10 and 3.32 Bq/g (average 2.04 Bq/g), corresponding to the average ^137^Cs inventory of 78.6 kBq/m^2^. The horizontal distributions of ^137^Cs concentrations and inventories in the surface soils are plotted in Figure 2. At first, we expected that ^137^Cs concentration or inventory would be higher near the water inlet if significant amounts of radiocesium had been transported into the paddy field through the irrigation water. We observed ^137^Cs within 5 cm of the surface because radiocesium input from the outside was expected to have accumulated on the very surface. However, such a clear trend was not observed in the distribution of the ^137^Cs concentration or inventory (Figure 2), suggesting that loading of radiocesium from the irrigation water was not significant. Quantitative evaluation of ^137^Cs input from irrigation water into the paddy field is discussed in the next section. The variation in the ^137^Cs distribution is partly attributed to the horizontal and vertical mixing of soils by plowing as mentioned above. 

### Input of irrigation water and loading of radiocesium on a paddy field

 The concentrations of radiocesium in irrigation water and suspended particles are listed in Table 1. The ^137^Cs concentrations in suspended particles of the 3 - 63 μm and > 63 μm fractions were 24.4 and 18.4 Bq/g, respectively, corresponding to 0.135 and 0.090 Bq/L. Cesium-137 concentrations in the soluble (i.e. < 0.45 μm) and total fractions were 0.074 and 0.302 Bq/L, respectively. The ratio of ^137^Cs in the 3 - 63 μm fraction to the total was the highest (45%); the second highest was 30% in the > 63 μm fraction. The contribution of the soluble fraction was also significant (25%). In contrast, the contribution of the 0.45 - 3 μm fraction was less than 1% and negligibly small.

**Table 1:** ^137^Cs concentrations of soluble and particle fractions in irrigation water.

		Sampling date	Measurement date	Particle concentration	^137^Cs^*^	^137^Cs^*^	Ratio
				(mg/L)	(Bq/g)	(Bq/L)	(%)
Paticle fraction	> 63 μm		January 21, 2013	4.92	18.4 ± 1.3	0.090 ± 0.007	30.0
	3 - 63 μm		January 28, 2013	5.54	24.4 ± 2.4	0.135 ± 0.013	44.7
	0.45 - 3 μm	August 31, 2012	January 28, 2013			0.0021 ± 0.0001	0.7
Soluble fraction	< 0.45 μm		January 7, 2013			0.074 ± 0.007	24.6
Total						0.302 ± 0.017	100

* Error was calculated as 1σ standard deviation from counting statistics.

 We evaluated the load of ^137^Cs into the paddy field using the results of paddy soil and irrigation water analysis. The average inventory of ^137^Cs within 5 cm of the surface was 78.6 kBq/m^2^, and the area of the paddy field is 740 m^2^. Consequently, the total amount of ^137^Cs in the uppermost 5 cm soil layers was estimated to be ca. 58,000 kBq. The surface of the paddy field is usually covered and filled with water during cultivation. We do not know accurately the volume of irrigation water that was drawn into the paddy field. If we assume that the height of water in the paddy field was 5 cm considering a typical water level, the field was filled with 37,000 L of irrigation water. Furthermore, if the ^137^Cs concentration in irrigation water was the same as in Table 1 and constant throughout cultivation, the total loading would be 11.2 kBq, which is much smaller than the 58,000 kBq of fallout ^137^Cs originally contained in the paddy field, as estimated from ^137^Cs concentration in the paddy soils. It should be noted that more than 50% of fallout ^137^Cs was distributed in soil layers below 5 cm (Table S1). From the calculation, we assess that the loading of radiocesium from irrigation water was negligible. Even if we assume further water intake with 500,000 L of irrigation water based on previous work [15], the loading (150 kBq) is still much smaller than the total from fallout. 

 We further considered input of radiocesium by rainfall events because rainfall possibly promotes erosion of surface soil in surrounding environments, preferentially fine particles with high radiocesium concentrations, resulting in a higher loading of radiocesium on a paddy field. We observed the load of suspended particles and radiocesium coming into the paddy field during a rainfall event from August 23 to September 19 (Table 2). The average concentration, over the rainfall event, of suspended sediment was calculated to be 231 mg/L based on the discharge of water and weight of suspended load. The ^137^Cs concentration in the suspended sediment was 31.8 Bq/g, corresponding to 1.26 kBq of ^137^Cs loading. The loading of ^137^Cs from the rainfall event is negligible relative to the total inventory of the paddy field (i.e. > 58,000 kBq).

**Table 2:** Loading of suspended sediment and ^137^Cs during a rainfall event.

Date Time	Rain fall	Discharge	SS load	^137^Cs	Load of ^137^Cs
	(mm)	(m^3^)	(g)	(Bq/g)	(kBq)
September 6 12:10-12:40	8.8	0.172	39.7	31.8	1.26

The estimation of ^137^Cs loading discussed above suggests that water intake from irrigation water during normal periods did not and will not influence the spatial distribution and inventory of ^137^Cs amounts in the paddy field. In addition, the loading of ^137^Cs from a rainfall event was fairly small. However, both the concentration of suspended sediment in irrigation water and ^137^Cs concentration in the suspended sediment during the rainfall event were higher than during a normal period (Tables 1 and 2). Consequently, to understand changes in ^137^Cs distribution and inventory, it is important to monitor ^137^Cs loading associated with rainfall throughout cultivation, considering the migration of ^137^Cs as particulate matters from the surrounding watershed to the paddy field as a sink. 

## Conclusions

 We investigated the spatial distribution of radiocesium vertically and horizontally in a paddy field to evaluate changes of distribution during cultivation. Disturbance of the original depth distribution of radiocesium indicates mixing of soil layers by plowing. The horizontal distribution of ^137^Cs did not show clear evidence of influence by irrigation water on the inventories. Our results of ^137^Cs analysis for the irrigation water coming into the paddy field showed that the amount of ^137^Cs newly added was negligibly small compared with the ^137^Cs originally contained in the paddy field. However, we note the possibility that loading of ^137^Cs on a paddy field can significantly increase if many heavy rainfall events occur during cultivation.

## Supporting Information

Table S1 **Depth distribution of 137Cs concentrations and inventories in soil core samples.**
(XLSX)

Table S2 **137Cs concentrations and inventories in surface soil samples.**
(XLSX)
